# Hsa_circ_0000418 promotes the progression of glioma by regulating microRNA-409-3p / pyruvate dehydrogenase kinase 1 axis

**DOI:** 10.1080/21655979.2022.2049027

**Published:** 2022-03-09

**Authors:** Zhihui Ma, Zhen Chen, Ying Zhou, Yanping Li, Shuyang Li, Hongxia Wang, Ji Feng

**Affiliations:** a Department of Neurosurgery, The First Hospital of Qinhuangdao, the First Hospital of Qinhuangdao, Qinhuangdao, Hebei, China; b Department of Anesthesiology, Qinglong Manchu Autonomous County Hospital, Qinhuangdao, Hebei, China; c Department of Anesthesiology, The Third Hospital of Qinhuangdao, Qinhuangdao, Hebei, China; d Supply Department, Qinglong Manchu Autonomous County Hospital, Qinhuangdao, Hebei, China; e Operating Room, Qinglong Manchu Autonomous County Hospital, Qinhuangdao, Hebei, China

**Keywords:** Circ_0000418, glioma, miR-409-3p, PDK1

## Abstract

Glioma is the commonest intracranial malignancy, and circRNAs are important regulatory factors which are implicated in the development of glioma. Nonetheless, the role of circRNAs in glioma is largely unknown. The research is performed to elaborate on the biological role of has_circ_0000418 (circ_0000418) in glioma progression and its potential molecular mechanism. The differentially expressed circRNAs in glioblastoma patient derived cells and neural progenitor cells were analyzed based on the microarray data of GSE146463. Additionally, qRT-PCR and Western blot experiments were conducted to measure the expression of circ_0000418, microRNA-409-3p (miR-409-3p) and pyruvate dehydrogenase kinase 1 (*PDK1*) in glioma tissues/cells. Cell growth and cell cycle distribution were monitored using CCK-8 assay, BrdU assay and flow cytometry. Bioinformatics prediction, dual-luciferase reporter gene experiment and RIP assay were conducted to verify the targeting relationship between circ_0000418 and miR-409-3p, miR-409-3p and PDK1 3ʹUTR. In this work, we observed that, circ_0000418 expression level was significantly up-regulated in glioma tissues and cell lines. Circ_0000418 overexpression facilitated glioma cell growth and accelerated cell cycle progression, while knockdown of circ_0000418 produced the opposite effects. Circ_0000418 specifically combined with miR-409-3p, and circ_0000418 negatively modulated the expression of miR-409-3p. PDK1 acted as a target gene of miR-409-3p, and PDK1 could be positively and indirectly modulated by circ_0000418 in glioma cells. In summary, circ_0000418 enhances glioma cell growth and accelerates cell cycle progression by regulating miR-409-3p/PDK1 axis.

## Graphical abstract

**Figure:**
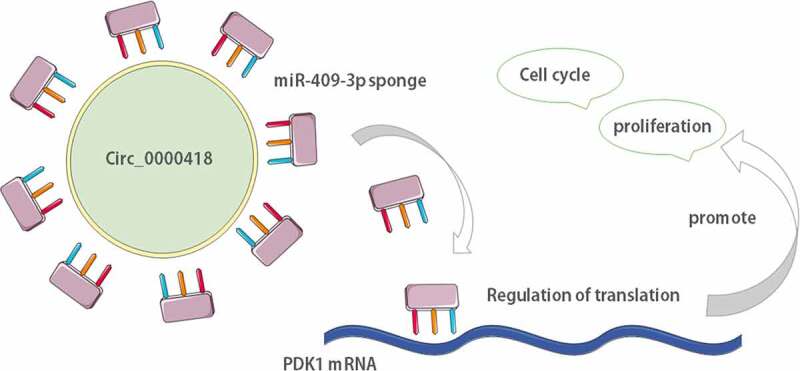

## 1 Introduction

Glioma is a malignant tumor which originates from neuroepithelial cells, taking up 40–50% of all intracranial malignancies, and is characterized by high morbidity, high relapse rate and high mortality [1]. The current treatments for glioma include surgical resection, adjuvant radiotherapy and chemotherapy, and targeted therapies, however, the average overall survival time of patients was only 12–18 months [2]. Therefore, the identification of new therapeutic targets for glioma is urgent.

Circular RNAs (circRNAs) are ncRNAs that have a closed-loop structure [2]. They are widely present and stably expressed in eukaryotic transcriptome [3]. Accumulating studies demonstrate that circRNAs are closely correlated with glioma tumorigenesis and development. For instance, circ-AKT3 is under-expressed in glioma tissues; circ-AKT3 overexpression restrains the growth, radioresistance and tumorigenicity of glioma cells [4]. Circ_0043278 is significantly overexpressed in glioma tissues and cells, and its high expression correlates with unfavorable prognosis of the patients; knockdown of circ_0043278 inhibits glioma cell growth, migration and invasion, and suppresses *in vivo* tumorigenesis [5]. Circ_0000418, a member of circRNAs, is involved in mediating the anti-depressant effect of *adenosine deaminase acting on RNA 1* [6]. In the present work, our bioinformatics analysis implies that circ_0000418 expression is up-regulated in glioblastoma patient derived cells. Nevertheless, the detailed expression pattern and biological function of circ_0000418 in glioma are rarely reported.

MiR-409-3p is down-regulated in different malignancies, and it can regulate cellular biological processes like growth, apoptosis, differentiation and migration by targeting different genes. For instance, miR-409-3p inhibits the aggressiveness of glioma cells by targeting *high mobility group nucleosome binding domain 5* (*HMGN5*) [7]. Pyruvate dehydrogenase kinase 1 (*PDK1*) belongs to the serine/threonine protein kinase family; PDK1 is significantly overexpressed in glioma tissues, and the knockdown of PDK1 restrains the colony formation ability of glioma cells [8,9]. The competitive endogenous RNA (ceRNA) theory states that circRNAs work as molecular sponge that regulate downstream target genes’ expression by sponging miRNAs [10]. Our bioinformatics analysis reveals that both of circ_0000418 and PDK1 3ʹUTR contain complementary binding sites with miR-409-3p. However, whether circ_0000418/miR-409-3p/PDK1 forms a ceRNA network in the tumorigenesis of glioma needs to be verified.

As mentioned above, in this work, we supposed that there was a ceRNA regulatory mechanism among circ_0000418, miR-409-3p and PDK1 in glioma progression. The goal of this work is to validate this hypothesis. This work showed that circ_0000418 was up-regulated in glioma tissues and cells, and circ_0000418 facilitated the growth and cell cycle progression of glioma cells by decoying miR-409-3p and up-regulating PDK1. This study hopefully provides an innovative molecular marker/target for the diagnosis and therapy of glioma.

## 2 Materials and methods

### 2.1 Sample collection

A total of 47 subjects who were diagnosed with glioma from June 2018 to December 2020 were selected. The surgically removed malignant tissues and matching adjacent tissues were collected and instantly preserved in liquid nitrogen. All subjects did not undergo chemotherapy, radiotherapy or other anti-cancer treatments before the surgery. This work was executed under the guidance of the Ethics Committee of the First Hospital of Qinhuangdao (Approval No. 20180305). All subjects signed an informed consent form before surgery.

### 2.2 Cell culture

Astrocyte cell line NHA and human glioma cell lines (U-87 MG, U-138 MG, U-118 MG, T98-G, LN-229, LN-18) were procured from ScienCell (San Diego, CA, USA) or American Type Culture Collection (ATCC) (Manassas, VA, USA), respectively. All cells were cultured in Roswell’s Park Memorial Institute 1640 (RPMI-1640) medium (Gibco, Carlsbad, CA, USA) containing 10% fetal bovine serum (FBS) (Gibco, Grand Island, NY, USA), 100 U/mL penicillin (Mediatech, Manassas, VA, USA) and 0.1 mg/mL streptomycin (Mediatech, Manassas, VA, USA) at 37°C with 5% CO_2_.

### 2.3 Cell transfection

Empty plasmid (negative control plasmid, NC), circ_0000418 overexpression plasmid (circ_0000418), si-NC, two circ_0000418 siRNAs (si-circ_0000418-1 and si-circ_0000418-2), miR-negative control (NC), miR-409 mimics, inhibitor-negative control (NC), miR-409-3p inhibitors (miR inhibitors) were procured from GenePharma Co., Ltd. (Shanghai, China). LN-229 cells and T98-G cells were planted into 24-well plats at 3 × 10^5^ cells/well and incubated at 37°C with 5% CO_2_ for 24 h before cell transfection. The above oligonucleotides/vectors were transfected into LN-229 and T98-G cells using Lipofectamine® 3000 (Invitrogen, Carlsbad, CA, USA) according to the protocols provided by the manufacturer. Transfection efficiency was measured by quantitative real-time PCR (qPCR) 48 h after the transfection.

### 2.4 qPCR

The RNA was extracted from tissues and cell lines by TRIzol reagent (Invitrogen, Shanghai, China), and after determination of concentration and purity, the total RNA was reverse transcribed into cDNA with the RevertAid RT kit (Thermo Fisher Science, Waltham, MA, USA). For the reverse transcription of miRNA, miRNA First Strand cDNA Synthesis Kit (Sigma-Aldrich, Louis, MO, USA) was used. Subsequently, the cDNA (1ng/μL) was used as the template for DNA amplification with the qRT-PCR assay kit (Stratagene, La Jola, CA, USA). Reaction system (20 μL): 2 μL of cDNA, 10 μL SYBR Green Mix (Takara, TX, USA), 0.4 μL of ROX Reference Dye, 0.8 μL of forward primer, 0.8 μL of reverse primer, 6 μL of ddH_2_O. The DNA amplification was performed on Applied Biosystems 7500 Real-time system (Applied Biosystems; Thermo Fisher Scientific, Inc., Foster City, CA, USA). PCR thermal cycling parameters were as follows: 95°C for 5 min, with a 3-step reaction: 94°C denaturation at 94°C for 30s and annealing at 60°C for 30s. 45 cycles were performed. Circ_0000418, miR-409-3p and PDK1 mRNA relative expressions were calculated using the 2^−ΔΔCt^ method using GAPDH and U6 as the internal references. The primer sequences are as follows:

hsa_circ_0000418 Forward: 5’-TGGAGTAAATCAACCAAAACGA-3’ and reverse 5’-TGTGCCCGCAATATTCATTA-3’.

miR-409-3p Forward: 5’-GAATGTTGCTCGGTGA-3’ and reverse 5’-GTGCAGGGTCCGAGGT-3’.

PDK1 mRNA Forward: 5’-CTGGCTGGATTTGGTTACGG-3’and reverse 5’-ACTCCGTTGACAGAGCCTTAAT-3’

GAPDH Forward: 5’-AATCCCATCACCATCTTCC-3’and reverse 5’-CATCACGCCACAGTTTCC-3’.

U6 forward: 5’-CGCTTCGGCAGCACATATAC-3’ and reverse 5’-AACGCTTCACGAATTTGCGT-3’.

### 2.5 Cell counting kit-8 (CCK-8) experiment

The transfected cells were inoculated into 96-well plates (2 × 10^3^ per well). 10 μL of CCK-8 reagent (Dojindo, Rockville, MD, USA) was then supplemented to each well at 24 h, 48 h, and 72 h and incubated for 2 h. The absorbance values of each well were appraised at 450 nm using a Multiskan™ spectrophotometer (Thermo Fisher Scientific, Grand Island, NY, USA).

### 2.6 BrdU assay

Briefly, LN-229 and T98-G cells were plated in 24-well plates at 2.5 × 10^4^ cells/well, and cultured for 24 h, followed by the addition of BrdU solution (Beyotime Biotechnology, Shanghai, China) to incubate for another 4 h. Then, the cells were fixed, and incubated with anti-BrdU antibody for 2 h, and then incubated with secondary antibodies for 1 h. The nucleus was marked by 10 μmol/L DAPI for 5 min. Ultimately, an upturned fluorescence microscope was utilized for observing the cells, and the percentage of BrdU-positive cells was calculated.

### 2.7 Flow cytometry

The transfected LN-229 and T98-G cells were fixed in 70% ethanol overnight at 4°C overnight. On the following day, the glioma cells were washed with phosphate buffer saline (PBS) and resuspended in 100 μL of PBS, and 5 μL of propidium iodide solution (PI, BD Bioscience, Franklin Lakes, NJ, USA) was added to stain the cells for 30 min. After the cells were washed by PBS again, a flow cytometer was employed to detect the cell cycles. FSC v.s. SSC figure was used to exclude the cell debris (R1); FSC v.s. PI figure was used to exclude the dead cells (R2). Then the cell cycle distribution was analyzed, with X-axis indicating the DNA content, and the Y-axis indicating the number of the cells.

### 2.8 Western blot assay

LN-229 and T98-G cells were lysed using RIPA buffer (Beyotime, Shanghai, China). A BCA Protein Assay Kit (Beyotime, Shanghai, China) was used to quantify the protein concentrations. 25 μg of protein sample in each group was separated by SDS-PAGE and then transferred into the polyvinylidene fluoride (PVDF) membrane. Afterward, the membranes were incubated with primary antibodies anti-PDK1 (ab202468, 1:1000, Abcam, Cambridge, UK) and anti-GAPDH (ab9485, 1:1000, Abcam, Cambridge, UK) overnight at 4°C. The membrane was cleaned 3 times for 5 min each time with tris buffered saline tween, and then incubated with the secondary antibody Goat Anti-Rabbit IgG H&L (ab150077, 1:2000, Abcam, Cambridge, UK) for 1 h at 37°C. Finally, the protein bands were visualized using the enhanced chemiluminescence (ECL) kit (Beyotime, Shanghai, China).

### 2.9 Dual‑luciferase reporter gene experiment

The sequences of circ_0000418 or 3ʹUTR of PDK1 including the wild type (WT) or mutated type (MUT) binding sites of miR-409-3p were amplified and inserted into the luciferase reporter plasmid psiCHECK2 (Promega, Madison, WI, USA) to construct wild-type circ_0000418 (WT circ_0000418), wild-type PDK1 (WT PDK1), mutant-type circ_0000418 (MUT circ_0000418) and mutant-type PDK1 (MUT PDK1) reporting vectors. The above vectors were co-transfected with miR-409-3p mimics or miR-409-3p inhibitors into LN-229 and T98-G cells, respectively. After 48 h of transfection, the luciferase activity was determined using the dual-luciferase Reporter Assay Kit (Promega, Fitchburg, WI, USA).

### 2.10 RNA immunoprecipitation (RIP) experiment

Following the protocols provided by the manufacturer, a Magna RIP Kit (Millipore, Billerica, MA, USA) was applied in the RIP experiment to validate the interaction between circ_0000418 and miR-409-3p. Briefly, the transfected LN-229 and T98-G cells were lysed in RIP lysis buffer, and the cell extracts were incubated with magnetic beads coated with anti-IgG or anti-Ago2. Then the immunoprecipitate was incubated with Proteinase K. The purified RNA was analyzed using qPCR to detect the enrichment of circ_0000418 and miR-409-3p in the immunoprecipitate.

### 2.11 Bioinformatics analysis

GSE146463 was downloaded from GEO database (https://www.ncbi.nlm.nih.gov/gds). This dataset includes the circRNA microarray data of glioblastoma cells (n = 8) and neural progenitor cells (n = 3). The data of GSE146463 was analyzed by GEO2R. The circRNAs with *P* < 0.05 and |log_2_ fold change| > 1 were regarded as the differentially expressed circRNAs. Additionally, Circular RNA Interactome database (https://circinteractome.nia.nih.gov) [11], StarBase (https://starbase.sysu.edu.cn) [12], TartgetScan 7.1 databse (http://www.targetscan.org/vert_71) [13] and miRWalk database (http://mirwalk.umm.uni-heidelberg.de) [14] were searched for the targeting relationship among circ_0000418, miR-409-3p and PDK1.

### 2.12 Statistical analysis

All statistical analysis was performed using GraphPad Prism Ver 8.0. D’Agostino & Pearson omnibus normality test was used to analyze the normality of the data. All of the data were presented as mean ± standard deviation (SD), and differences analysis was conducted using Student’s *t*-test or one-way ANOVA. Correlations between the data were analyzed by Pearson’s correlation analysis. *P* < 0.05 denoted statistical significance.

## 3 Results

The goal of this work was to explore the differentially expressed circRNAs in glioma progression. Bioinformatics helps us obtain a up-regulated circRNA in glioma, circ_0000418. Then we used a series of experiments to investigate its expression characteristics in glioma tissues and cell lines, its function in the growth and cell cycle of glioma cells, and its downstream mechanism.

### 3.1 Circ_0000418 is highly expressed in glioma tissues and cell lines

**Figure 1.**
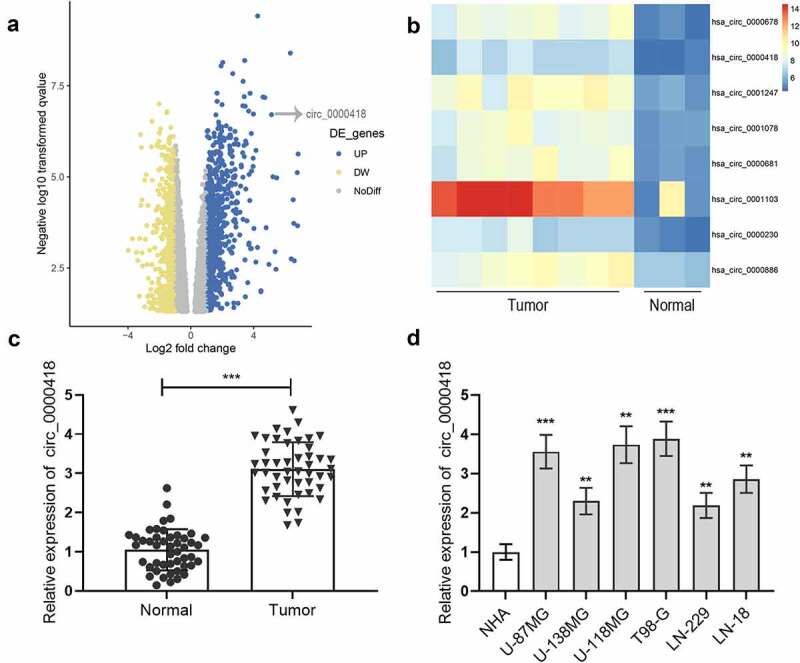
Circ_0000418 expression is significantly up-regulated in glioma a. The microarray dataset GSE146463 was downloaded from GEO database to analyze the differentially expressed circRNAs between glioblastoma patient derived cells and neural progenitor cells, and the volcano plot showed these circRNAs. The yellow indicated the down-regulated circRNAs, and the blue indicated the up-regulated circRNAs. b. Heat map was generated to show 8 representative up-regulated circRNAs in glioma tissues. c. Circ_0000418 expression in 47 cases of glioma tissues and normal tissues was measured by qPCR. d. Circ_0000418 expression levels in NHA cells and glioma cells (U-87 MG, U-138 MG, U-118 MG, T98-G, LN-229, LN-18) were detected by qPCR. ** *P* < 0.01, *** *P* < 0.001.

To probe circ_0000418 expression in glioma, the microarray dataset GSE146463 was accessed from the GEO database to examine the differentially expressed circRNAs between glioblastoma patient derived cells and neural progenitor cells. The results suggested that 540 circRNAs were down-regulated and 823 circRNAs were up-regulated in glioma (Figure 1(a)), and a heat map of 8 up-regulated circRNAs was generated (Figure 1(b)). Among these, circ_0000418 was chosen for the subsequent investigation. qPCR was conducted to detect circ_0000418 expression in tumor tissues and non-cancerous tissues of 47 glioma patients. The data implied that circ_0000418 expression was remarkably up-regulated in glioma tissues (*P* < 0.001) (Figure 1(c)). Also, circ_0000418 expression in glioma cells (U-87 MG, U-138 MG, U-118 MG, T98-G, LN-229, LN-18) was also significantly up-regulated compared to that in NHA (*P* < 0.01) (Figure 1(d)).

### 3.2 Circ_0000418 promotes glioma cell growth, and cell cycle progression

**Figure 2.**
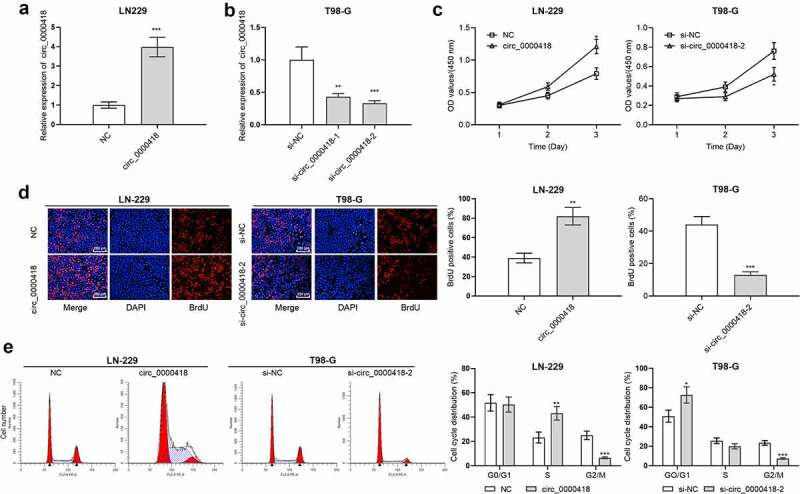
The biological function of circ_0000418 in glioma a-b. qPCR was carried out to detect the transfection efficiency of circ_0000418 overexpression plasmid and two circ_0000418 siRNAs. c-d. The effects of overexpression or knockdown of circ_0000418 on the growth of LN-229 and T98-G cells were analyzed using CCK-8 assay (c) and BrdU assay (d). e. Flow cytometry was executed to detect the effect of overexpression or knockdown of circ_0000418 on the cell cycle progression of LN-229 and T98-G cells. * *P* < 0.05, ** *P* < 0.01, *** *P* < 0.001.

Among the six glioma cells, circ_0000418 expression level was the lowest in LN-229 cells and the highest in T98-G cells. Hence, circ_0000418 overexpression plasmid and circ_0000418 siRNAs were transfected into LN-229 cells and T98-G cells, and we successfully constructed circ_0000418 overexpression and knockdown models (Figure 2(a,b)). CCK-8 experiment, BrdU experiment and flow cytometry showed that circ_0000418 overexpression promoted LN-229 cell growth and accelerates the cell cycle as opposed to the NC group (*P* < 0.05) (Figure 2(c-e)). Contrariwise, in T98-G cells, the knockdown of circ_0000418 suppressed glioma cell growth and cell cycle progression (Figure 2(c-e)).

### 3.3 Circ_0000418 directly targets miR-409-3p

**Figure 3.**
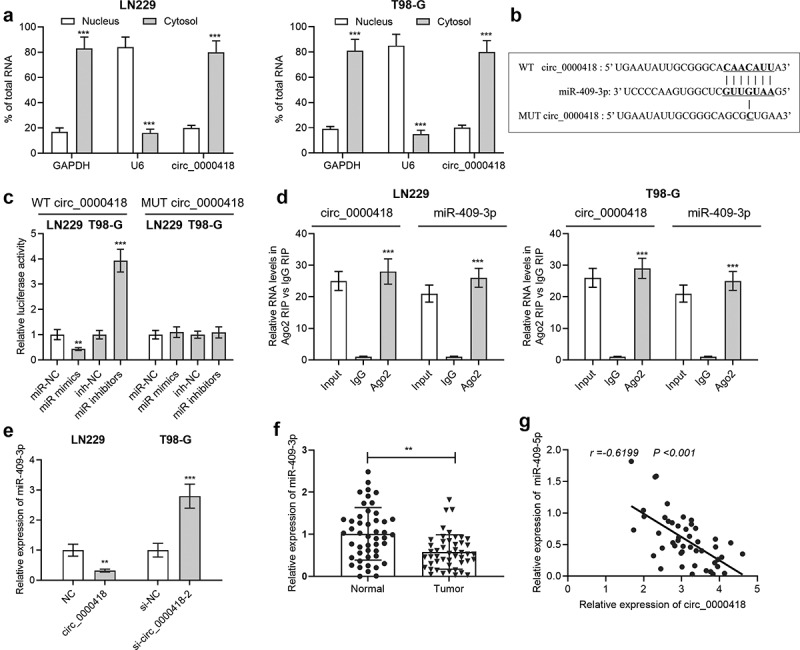
Circ_0000418 directly targets miR-409-3p a. The subcellular location of circ_0000418 in LN-229 and T98-G cells was evaluated by qPCR after that the nuclear and cytoplasmic fractions of glioma cells were isolated. b. The Circular RNA Interactome online database was employed to predict the binding site of circ_0000418 to miR-409-3p. c. WT circ_0000418 and MUT circ_0000418 were co-transfected with miR mimics or miR inhibitors into LN-229 and T98-G cells, respectively, and the dual-luciferase reporter gene system was employed to measure luciferase activity. d. The interaction between circ_0000418 and miR-409-3p was further validated using RIP assay. e. The effect of overexpression/knockdown of circ_0000418 on miR-409-3p expression in glioma cells was examined by qPCR. f. MiR-409-3p expression in 47 cases of glioma tissues and normal tissues was examined by qPCR. g. Pearson correlation analysis was performed to analyze the correlation between miR-409-3p expression and circ_0000418 expression in glioma tissues. ** *P* < 0.01, *** *P* < 0.001.

To validate the targeting correlation between circ_0000418 and miR-409-3p, the subcellular localization of circ_0000418 in glioma cells was identified using qPCR. The data implied that circ_0000098 was primarily found in the cytoplasm (*P* < 0.001) (Figure 3(a)). MiRNAs that can interact with circ_0000418 were predicted by Circular RNA Interactome, a possible binding site between circ_0000418 and miR-409-3p was discovered (Figure 3(b)). To validate the targeting relationship between the two, the dual-luciferase reporter gene assays were performed. Transfection with miR-409-3p mimics significantly inhibited the luciferase activity of wild-type circ_0000418 reporter (*P* < 0.01), while there was no remarkable alteration in the luciferase activity for mutated circ_0000418 reporter (*P >* 0.05) (Figure 3(c)). Furthermore, RIP assays validated that circ_0000418 and miR-409-3p were enriched by the Ago2-antibody compared with control IgG (*P* < 0.001) (Figure 3(d)). Furthermore, circ_0000418 overexpression was unveiled to substantially impede miR-409-3p expression in LN-229 cells, while in T98-G cells (*P* < 0.01), knockdown of circ_0000418 caused an increase in miR-409-3p expression (*P* < 0.001) (Figure 3(e)). Notably, miR-409-3p expression was markedly down-regulated in glioma tissues compared with that in non-cancerous brain tissues (*P* < 0.01) (Figure 3(f)), and circ_0000418 expression was negatively correlated with miR-409-3p expression in glioma tissues (r = −0.6199, *P* < 0.001) (Figure 3(g)).

### 3.4 Circ_0000418 participates in regulating the biological behaviors of glioma cells by sponging miR-409-3p

**Figure 4.**
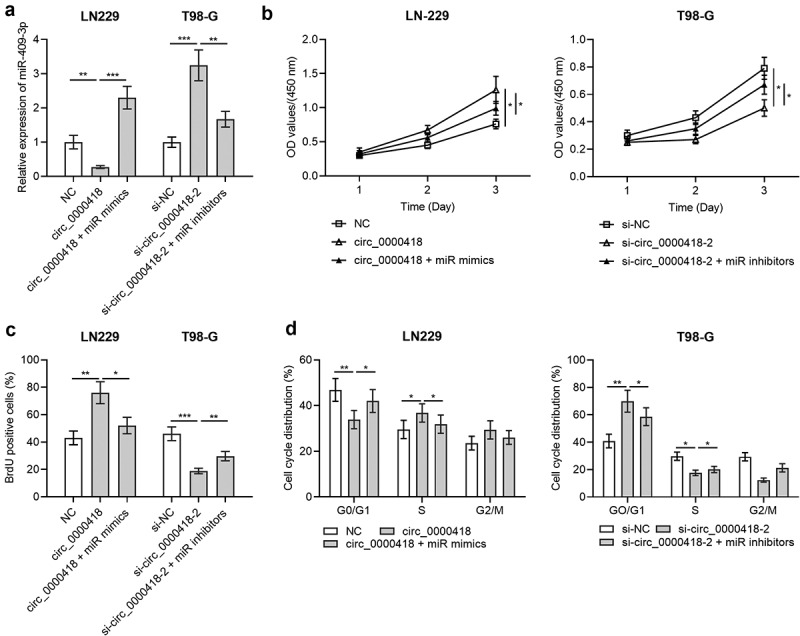
Effects of Circ_0000418 and miR-409-3p on the biological functions of glioma cells a. The transfection efficiency of circ_0000418 overexpression plasmid+miR mimics and si-circ_0000418-2+ miR inhibitors in LN-229 and T98-G cells was examined by qPCR. B-C. CCK-8 assay (b) and BrdU experiment (c) were applied to detect the effects of circ_0000418+ miR-409-3p mimics or si-circ_0000418+ miR-409-3p inhibitor on cell growth. d. The effect of circ_0000418+ miR-409-3p mimics or si-circ_0000418+ miR-409-3p inhibitor on cell cycle was examined by flow cytometry. * *P* < 0.05, ** *P* < 0.01, *** *P* < 0.001.

To probe the role of miR-409-3p in the progression of glioma, circ_0000418 overexpression plasmid+miR-409-3p mimic and si-circ_0000418-2+ miR-409-3p inhibitors were co-trasfected into LN-229 cells and T98-G cells, respectively. qPCR manifested that the transfection was successful (Figure 4(a)). Subsequently, the CCK-8 experiment, BrdU experiment and flow cytometry showed that compared with the circ_0000418 group, co-transfection of miR-409-3p mimics into LN-229 cells significantly inhibited cell growth and blocked the cell cycle progression (*P* < 0.05) (Figure 4(b-e)). On the other hand, compared with si-circ_0000418-2 alone, co-transfection of si-circ_0000418-2 and miR-409-3p inhibitors into T98-G cells promoted cell growth and facilitated the cell cycle progression (*P* < 0.05) (Figure 4(b-e)).

### 3.5 Circ_0000418 up-regulates PDK1 expression through repressing miR-409-3p

**Figure 5.**
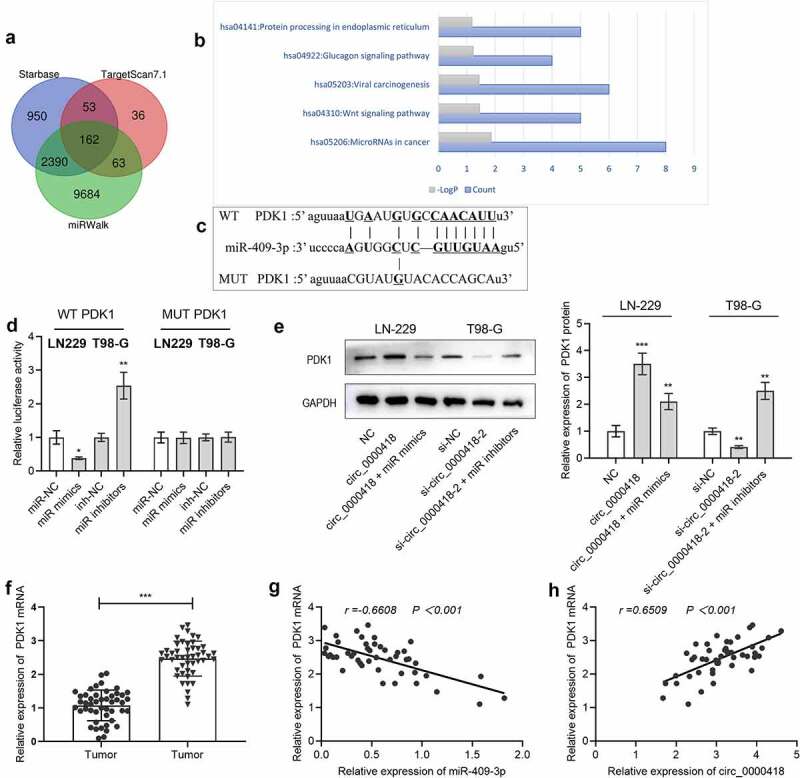
Circ_0000418 up-regulates PDK1 expression through adsorption of miR-409-3p a. The downstream targets of miR-409-3p were predicted through bioinformatics websites StarBase, TargetScan and miRWalk. b. Enrichment analysis of downstream target genes of miR-409-3p was conducted using the KEGG database. c. The binding site between miR-409-3p and PDK1 mRNA 3ʹUTR. d. WT PDK1 and MUT PDK1 were co-transfected with miR-409-3p mimics and miR-409-3p inhibitors into 293 T cells, and then the luciferase activity was evaluated. e. Western blot was applied to detect the effects of circ_0000418 and miR-409-3p on PDK1 expression. f. qPCR was implemented to detect PDK1 mRNA expression in 47 pairs of glioma tissues and non-cancerous brain tissues. G-H. Pearson correlation analysis was performed to analyze the correlation between circ_0000418, miR-409-3p and PDK1 mRNA expression in glioma tissues. * *P* < 0.05, ** *P* < 0.01, *** *P* < 0.001.

To look for the downstream targets of miR-409-3p, StarBase, TartgetScan 7.1 and miRWalk databases were utilized to screen the candidate targets of miR-409-3p, and 162 genes including PDK1 were unearthed to be the candidate targets of miR-409-3p (Figure 5(a)). Additionally, the KEGG database was utilized to conduct pathway enrichment analysis on the potential downstream target genes of miR-409-3p, and the results suggested that the above target genes were correlated with the triggering of the Wnt signaling pathway (Figure 5(b)). To validate whether miR-409-3p could bind with PDK1 mRNA 3’-UTR, a luciferase reporter experiment was implemented. The data showed that transfection of miR-409-3p mimics remarkably inhibited the luciferase activity of the 3’-UTR of wild-type PDK1, and transfection of miR-409-3p inhibitors remarkably increased the luciferase activity of the 3’-UTR of wild-type PDK1 (*P* < 0.05), while the two had no significant effects on the luciferase activity of MUT-PDK1 (*P* > 0.05) (Figure 5(c,d)). Additionally, the data of Western blot also manifested that circ_0000418 overexpression significantly promoted PDK1 protein expression (*P* < 0.001), while co-transfection of miR-409-3p mimics weakened this effect (*P* < 0.01); knockdown of circ_0000418 significantly inhibited PDK1 protein expression (*P* < 0.01), while suppression of miR-409-3p reversed this effect (*P* < 0.01) (Figure 5(e)). Meanwhile, qPCR presented that PDK1 mRNA was substantially overexpressed in tumor tissues (*P* < 0.001) (Figure 5(f)). In addition, miR-409-3p and PDK1 mRNA expressions were negatively correlated (r = −0.6608, *P* < 0.001), while circ_0000418 and PDK1 mRNA expressions were positively associated (r = 0.6509, *P* < 0.001) (Figure 5(g,h)).

## 4 Discussion

CircRNAs are widely present in mammalian cells [3], affecting different biological activities, including cell growth and differentiation. Accumulating studies have shown that circRNA is differentially expressed between tumor tissues and corresponding paracancerous tissues, and can be employed as novel biomarkers and potential therapeutic targets for diverse malignancies [15,16]. In glioma. Circ-FBXW7 is downregulated in gliomas and correlated with shorter overall survival time of the patients [17]. Circ_01844 expression is down-regulated in glioma tissues and cells, and circ_01844 overexpression significantly impedes glioma cell growth and migration and induces cell apoptosis [18]. Circ-EPB41L5 is significantly under-expressed in glioma tissues and cells, and is closely associated with poor prognosis of the patients; functionally, circ-EPB41L5 inhibits glioma cell growth, colony formation, migration and invasion [19]. Circ_0000418 is much less investigated. A previous study reports that circ_0000418 is associated with the pathogenesis of depression [6]. In this research, for the first time, the expression feature and biological function of circ_0000418 in cancer biology is investigated. Circ_0000418 expression was revealed to be up-regulated in glioma tissues, and functional studies unveiled that circ_0000418 remarkably enhanced cell growth and facilitated the cell cycle progression of glioma cells, implying that circ_0000418 is a potential molecular marker for glioma and a possible therapeutic target.

MiRNA is non-coding small molecule RNA of 18–25 nucleotides in length [20]. MiRNAs regulate gene expression via binding to the 3’-UTR of target mRNAs, and they modulate about 30% of human protein-coding genes [21]. MiRNAs are implicated in diverse biological activities, and they are also involved in the tumorigenesis of human malignancies [22–26]. Reportedly, miR-409-3p is remarkably under-expressed in cervical cancer tissues and cells, and miR-409-3p overexpression specifically down-regulated the expression level of *activating transcription factor 1*, inhibiting cell growth, migration, invasion and glycolysis [27]. MiR-409-3p is also under-expressed in breast cancer tissues and cells and correlates with TNM stage, lymph node metastasis and short patient survival [28]. In addition, the role and mechanism of miR-409-3p in glioma have been reported in some previous studies. For example, in glioma, miR-409-3p under-expression is associated with poor prognosis of the patients [29]. Additionally, miR-409-3p is significantly under-expressed in glioma and miR-409-3p impedes cell growth and invasion by negatively regulating *HBGNBD5* [7]. Similarly, in the current work, it was found that miR-409-3p was significantly under-expressed in glioma tissues and cells, and we also demonstrated that circ_0000418 promoted glioma cell growth and promoted the cell cycle progression by specifically down-regulating miR-409-3p.

PDK1 is a serine/threonine-protein kinase with a size of 67 KD, which induces the phosphorylation of pyruvate dehydrogenase to regulate the homeostasis of carbohydrate fuels; PDK1 is vital in regulating a lot of biological processes such as cell growth, differentiation and apoptosis [9]. Importantly, PDK1 is highly expressed in cancers including breast cancer, non-small cell lung cancer and pancreatic cancer, and it functions as an oncogene [30–32]. Notably, PDK1 promotes glioma cell migration and invasion *in vitro* and glioma xenograft growth *in vivo* through up-regulation of c-Jun protein and induction of epithelial-mesenchymal transition [33]. Another study reports that PDK1 expression is up-regulated in glioma tissues and cells; silencing of *PDK1* expression led to reduced level of lactate and ATP, accumulation of ROS, mitochondrial damage, reduced cell growth, and cell apoptosis [34]. PDK1 has been reported to be modulated by serveral miRNAs. A previous study identifies *PDK1* as a downstream target of miR-128-3p in glioma cells [34]. Additionally, *PDK1* is validated to be a target gene of miR-454 in glioma, and miR-454 overexpression significantly inhibits PDK1 expression, thereby suppressing cell growth and blocking the cell cycle in G0/G1 phase [35]. In the research, we found that circ_0000418 could decoy miR-409-3p to up-regulate PDK1 expression, which provides a novel explanation of PDK1 dysregulation in glioma cells.

## 5 Conclusion

Taken together, this work found that circ_0000418 expression was up-regulated in glioma tissues and cells. Circ_0000418 facilitated glioma cell growth, and accelerated the cell cycle by targeting the miR-409-3p/PDK1 axis, indicating that circ_0000418 may be a potential diagnostic marker and a new therapeutic target for glioma, which provides a theoretical basis for the clinical therapy of glioma.
